# Is pharmacogenomics a reality? Challenges and oppurtunities for India

**DOI:** 10.4103/0971-6866.80350

**Published:** 2011-05

**Authors:** Moinak Banerjee

**Affiliations:** Human Molecular Genetics Laboratory, Rajiv Gandhi Center for Biotechnology, Trivandrum, Kerala, India

The global pharmaceutical market is approximately US$ 825 billion. While we evaluate the volume of the market, it is also important to know that which aspect of the drug response this market size refers to. The term drug response includes two facets: drug effectiveness (efficacy) and drug side effects. It is estimated that, on average, as much as 40% of the medicines that individuals take every day are not effective. In fact, for certain medications the estimate of non effectiveness is well over 50%. This would mean that close to US$ 400 billion are effectively wasted. While referring to specific classes of drugs, a conservative estimate indicates that 15-35% of patients have an adequate or no response to b-blockers; 7-28% of patients have an adequate or no response to angiotensin-converting enzyme inhibitors; 9-23% of patients respond inadequately to selective serotonin reuptake inhibitors; 20-50% of patients have an adequate or no response to tricyclic antidepressants, 10-20% of patients do not initially respond to antipsychotic therapy, and an additional 20-30% who do respond early on eventually relapse and some develop serious side effects.[1] In more than 91.2% of drug treatments approved in the USA in the last decade, the teratogenic risks in human pregnancy remain undetermined.[2] Evaluating the side effects of the drugs would further add burden to the economy of drug response. These observations raise serious apprehensions about how India should address its health benefit concerns. India occupies 20% of the world population, but shares only 2% of the global pharmaceutical market and that too for generic drugs. This would mean that newer and probably safer drugs are out of reach of common Indians. Adverse drug reporting is also not a common practice in most of the clinical establishments in India.

As early as 1892, Sir William Osler made an observation that “If it were not for the great variability among individuals, medicine might as well be a science and not an art.” Today the science behind this art is being dissected out by understanding the pharmacogenomic variability. In a recent observation it has been very clearly demonstrated that how genomic information can yield useful and clinically relevant information for individual patients.[3] Now, it has been established that the economic burden of drug response can be drastically minimized by genomic technologies.[4] Realizing the potential of pharmacogenomic profiling, the top 10 global pharmaceutical giants created a public SNP database as early as 1999, through an industry-funded industry academia US nonprofit organization and named it “The SNP Consortium Ltd.” The objective of this consortium was to develop a high-density, high-quality map with shared financial risks and less duplication of effort.[5] The consortium initiated the program with a budget of US$48 million and has already earned back their investment by providing access to nonpartners of the consortium through their database. This joint venture became a model for streamlining the process of drug response monitoring in clinical trials. Pharmacogenomics Knowledgebase (www.PharmGKB.org) initiated a mission to collect, encode, and disseminate knowledge about the impact of human genetic variations on drug response. Subsequently, FDA in 2005 incorporated the guidelines for genomic data submission for drug approval. Presently, the genomic data submission for drug approval is voluntary but with increasing awareness very soon this would be mandatory. Today there are several FDA approved drugs with pharmacogenomic information in their labels (www.fda.gov/Drugs/ScienceResearch/ResearchAreas/Pharmacogenetics/ucm083378.htm). Based on patient’s genotype, gender, age, family, and medical history, more than 650 drug-related variants have been identified for their clinical relevance.[6] With rapid growth of IT infrastructure and penetration of Internet technologies, these information are fast reaching the Indian household. This may drastically change the practice of medicine in coming years and may cause concern for practitioners for being unaware of the developments. Since Indians live in a strong emotionally and culturally driven society, implications can be wide ranging.

The country should debate on how India can channel its limited financial resources, knowing that the drug discovery programs and the drug response monitoring programs can be of huge economic liabilities. The challenges are further aggravated by the genetic diversity of the resources for both, the natural product drug discovery programs and drug response monitoring programs. Knowing that the drug discovery programs are full of uncertainties,[7] in contrast, the drug response monitoring is always a result-oriented initiative. Interestingly, India’s pharmaceutical market, mostly deals with generic drugs, therefore, it further strengthens the view that drug response monitoring program based on pharmacogenomic profiling of Indian populations is ideal for having a safer response to medications. For pharmaceutical companies worldwide, India is not only a potentially huge market for drug therapeutics but is also a repository of important human genetic diversity. Understanding this diversity is valuable because it better defines those population subgroups that will benefit more from a particular drug than others, and allows the detection of side-effects that might not be seen in populations that are mainly Caucasian. On a similar note, it has been argued that the blockbuster analgesic drug Vioxx (rofecoxib) which was withdrawn from the market because of its cardiovascular complications in those who took it for more than 18 months could have been put to use in populations with a safer pharmacogenomic background.[8] These pharmacogenomic variations have evolutionary origins. This suggests that not every individuals need to be screened, but one need to understand the evolutionary history of individuals and its role in establishment of various cultural groups. Several genetic studies have been carried out to understand the Indian population history and migrations. The Indian populations are stratified into different population groups based on religion, hierarchical structure of different castes, or subgroups in various religions which in turn are further stratified based on linguistic affiliations. Historical gene flow into India has also contributed to a considerable obliteration of genetic histories of contemporary populations.[9] A recent study provided strong evidence for two genetically divergent ancient populations that are ancestral to most present day Indians. One, the “Ancestral North Indians” (ANI), is genetically close to Middle Easterners, Central Asians, and Europeans, whereas the other, the “Ancestral South Indians” (ASI), is as distinct from ANI and East Asians as they are from each other.[10] Demic diffusion of the ancestral local progressive communities with the migrant communities has been hypothesized for the process of admixtures among Dravidian communities.[11] India conceptualized its first consortium based human genome variation database with an objective to catalog the variations in nearly 1000 candidate genes related to diseases and drug responses for predictive marker discovery, founder identification and also to address questions related to ethnic diversity, migrations, extent, and relatedness with other world population.[12] The knowledge gained from these studies and several individual-based studies can provide tremendous impetus to understanding of therapeutic response to drugs that Indians routinely take.

India is far behind in addressing the foreseeable challenge of drug response monitoring or even on biomarker discovery. It is indeed high time that we realizes the potential of pharmacogenomic technologies or we end up paying SNP Consortium Ltd. or Pfizer or AstraZeneca for accessing our own databases, as these companies are already in the process of screening the Asian-Indian subgroups living in the United States. Over the years, the genome technologies have drastically reduced the cost of genome sequencing. The first human genome sequence cost US$ 2.7 billion. Now, with next-generation rapid sequencing technology, a human genome can be sequenced for less than US$ 10,000, and in the foreseeable future, the cost could reach US$ 1000 while global pharmacogenomic testing of an individual could be less than US$ 100. Thus genomic technologies seems to have defeated the Moore’s law of computing and this clearly suggests that pharmacogenomic profiling would be affordable to every Indians in near future. In a recent survey in US indicated that more than 90% of Americans prefer pharmacogenomic profiling for drug dosing and drug selection.[13] Scientific journal, *Nature*, in 2010 indicated that India is way behind in the global map of genomic technology landscape. It is an opportunity for India to tap its intellectual resources to initiate a mission mode program in addressing the concerns of human health. The various genomic initiatives could also provide the foundation for approaching the future clinical trials possibly as early as, at the preclinical trial stage. This might minimize the failure rate in the early stages of drug discovery programs and could also result in reducing the economic burden of drug discovery programs. Even those drugs which might have failed in the early stages of clinical trials could be revived to test in different pharmacogenomic background of cell lines, animal models, or human populations. It might also open up avenues for generating pharmacogenomic variant specific *in vitro* screens. The present special issue on pharmacogenomics deals with some of these issues.
